# Hybrid office work in women and men: do directly measured physical behaviors differ between days working from home and days working at the office?

**DOI:** 10.1093/annweh/wxad057

**Published:** 2023-10-03

**Authors:** Viktoria Wahlström, Leticia Bergamin Januario, Svend Erik Mathiassen, Marina Heiden, David M Hallman

**Affiliations:** Department of Public Health and Clinical Medicine, Section of Sustainable Health, Umeå University, SE-901 87 Umeå, Sweden; Department of Occupational Health Science and Psychology, Centre for Musculoskeletal Research, University of Gävle, SE-801 76 Gävle, Sweden; Department of Occupational Health Science and Psychology, Centre for Musculoskeletal Research, University of Gävle, SE-801 76 Gävle, Sweden; Department of Occupational Health Science and Psychology, Centre for Musculoskeletal Research, University of Gävle, SE-801 76 Gävle, Sweden; Department of Occupational Health Science and Psychology, Centre for Musculoskeletal Research, University of Gävle, SE-801 76 Gävle, Sweden

**Keywords:** covid-19 pandemic, compositional data analysis, remote work, sedentary, temporal patterns, time in bed

## Abstract

**Objective:**

We investigated and compared temporal sitting patterns among male and female hybrid office workers when working at the office (WAO), working from home (WFH), and for non-working days (NWD).

**Methods:**

We analyzed data collected in 2020 among 165 hybrid office workers, carrying thigh-worn accelerometers for 938 days in total. Day type (WAO, WFH, or NWD) and time in bed were identified using diaries. Time awake was exhaustively classified as non-sitting time and time sitting in short, moderate, and long bouts. Effects of day type and gender on the 24-h compositions of physical behaviors were analyzed using multilevel linear mixed models.

**Results:**

During workdays (both WAO and WFH), workers spent less time in bed and more time sitting, particularly in moderate and long bouts, than during NWD. Time in bed was longer when working from home than when working at the office, and more of the awake time was spent sitting. Differences between WAO and WFH in ratios between short, moderate, and long bouts of sitting were small and inconsistent. Men spent more time sitting than women, and more time in moderate and long sitting bouts relative to short bouts.

**Conclusions:**

When working from home, hybrid office workers sat more during their hours awake compared to when working at the office. Sitting time was larger during working days than during non-working days and was higher in men than in women. These results may contribute to support organizational policies for hybrid work.

**What’s Important About This Paper?**

With increased hybrid work, it is important to understand activity patterns and their implications for work organization policies. This study found that office workers spent more time in bed and were sitting more during their time awake on days working from home compared to days working at the office. The distribution of sitting time between short, moderate, and long uninterrupted bouts did not differ between work locations. Sitting time was higher during working days compared to non-working days and men sat more than women.

## Introduction

Time spent sitting is an important determinant of health (Ekelund et al. 2019). Excessive sitting time is associated with increased risks for a range of non-communicable diseases, such as cardiovascular diseases, diabetes type 2, and mortality (Ekelund et al. 2019; Katzmarzyk et al. 2019). Office workers may be at a particular risk since both time at work and leisure entail extensive sitting (Healy et al. 2016; Danquah et al. 2017; Hallman et al 2018). During the COVID-19 pandemic, many office-based organizations recommended and implemented work from home (WFH) in order to reduce exposure to the corona virus and thus decrease the risk of infection (Eurofound 2020). Social restrictions differed between countries, and Sweden had less restrictive policies than most other European countries, avoiding a full-scale lockdown by relying on individuals to take personal responsibility (Eurofound 2020).

In Sweden hybrid work, in which working at the office (WAO) is combined with WFH in different proportions, became more common during the COVID-19 pandemic compared to before, and it will likely remain a predominant feature in many office-based occupation (Hickman et al. 2020). Swedish statistics show that about 45% of the workforce practiced hybrid work in May 2022, and about 15% worked from home at least half of their working days (Statistics Sweden 2022). For some workers, WFH may lead to higher flexibility and autonomy in how time is used, less time spent commuting, better performance, and an improvement in work–life balance (Eurofound 2020; Chafi et al. 2021). However, it can also lead to challenges with respect to working conditions and physical behaviors, for instance due to poor ergonomic conditions in home settings, and the likelihood of office workers performing computer-based work to a larger extent when working from home (Loef et al. 2022; Yang et al. 2023). A recent review, studying the influence of WFH on sitting and physical activity report that sitting time increased and physical activity at different intensity levels decreased (Wilms et al. 2022). Accordingly, a large prospective study on self-reported physical behaviors and hybrid work during the COVID-19 pandemic found that workers reported to be more physically inactive and sedentary to a larger extent when working from home during the pandemic than before, compared to workers who were still located at the office, and that workers WFH also reported more pain in the upper back, neck, shoulder, and arm than workers WAO, mediated by increased sedentariness (Loef et al. 2022). These results even applied to hybrid workers, but to a smaller extent.

The health effects of sitting depend not only on the total amount of time spent sitting, but also on the temporal pattern in which sitting is accumulated. Prolonged sitting may lead to negative effects on glycemic control compared to sitting in shorter bouts (Dempsey et al. 2017; Winkler et al. 2018) and to increased musculoskeletal pain (Coenen et al. 2017; Baker et al. 2018). A recent intervention study confirmed that more variation in sitting, in terms of a higher frequency of active breaks or postural shifts, reduced the incidence of new onsets of neck and low-back pain among office workers (Waongenngarm et al. 2021).

Previous studies have indicated that when WAO, men appear to sit more than women (Nooijen et al. 2018; Johansson et al. 2020). The difference may, to a large extent, be due to men and women having different work tasks, and this, in turn, may also affect differences between men and women when WFH. For instance, Appel-Meulenbroek et al. (2022) found that men with communication as an important component of their work were more inclined to WAO, while women, especially those who had administrative roles or work tasks with high concentration demands, preferred to WFH. Understanding the effect of gender on time spent sitting is important for understanding possible gender inequalities at and outside work, and thus for informing interventions. Gender differences in tasks during work as well as non-work may contribute to differences in sitting patterns, but the influence of gender on behaviors in hybrid workers during workdays (WAO and WFH) and non-working days is not known. Thus, it is of interest to investigate sitting patterns during both WAO and WFH for men and women separately.

So far, most studies investigating physical behaviors, including sitting, have used self-reported data, but self-reports may be inaccurate (Gupta et al. 2018; Wahlström et al. 2022). In particular, self-reports cannot be used to validly assess the temporal pattern of sitting, for instance how sitting is accumulated in periods of different durations throughout the day (Holtermann et al. 2017). Several studies have measured sitting among office workers using accelerometers (Bergman et al. 2018; Renaud et al. 2020) and many have even reported variables reflecting temporal patterns (Johansson et al. 2020; Larisch et al. 2021), but only few and small studies have investigated the effects of WFH on sitting patterns using objective methods (Hallman et al. 2021; Waongenngarm et al. 2021; Widar et al. 2021). In a pre-pandemic study of 23 Swedish office workers in academia, Widar et al (2021) found no differences in total sitting time between WAO and WFH, while participants more often switched between sitting and standing when WFH. A Swedish study (Hallman et al. 2021) using thigh-worn accelerometers among 27 office workers during the COVID-19 pandemic reported that WFH did not differ from WAO in the relative distribution of work time spent sitting, standing, and moving, while workers slept more when WFH. A study of 11 Brazilian university employees, also using thigh-worn accelerometers, found workers to spend more time in bed on workdays during the pandemic when they were required to WFH, and less time in physical activity of moderate and vigorous intensity relative to light-intensity physical activity, compared to pre-pandemic conditions (Brusaca et al. 2021). A study comparing Brazilian and Swedish office workers suggested, however, that physical behaviors during the pandemic may have differed considerably between countries (Brusaca et al. 2022). Since the knowledge regarding directly measured physical behaviors and their temporal patterns among office workers in hybrid arrangements is sparse and partly conflicting, there is a need for more and larger studies.

The primary aim of the present study was to document and compare physical behaviors, with a particular focus on sitting patterns, among Swedish hybrid office workers during days working at the office and days working from home during the COVID-19 pandemic. Behaviors were addressed in a 24-h perspective, including time in bed (as a proxy for sleep), sitting (distributed in periods of different durations), and non-sitting. These behaviors were even assessed during non-working days, as a reference. A second aim was to determine the extent to which this time use pattern differed between women and men. The intention of the study was to provide evidence supporting recommendations regarding hybrid work even after the COVID-19 pandemic.

## Method

### Design, study population, and recruitment

We analyzed cross-sectional data from the FLOC cohort, which aims at investigating opportunities and challenges in flexible work (Svensson et al. 2022). Data were collected during 2020 in office workers in 3 organizations in mid-Sweden, i.e. one large industrial company (data collected October–December 2020) and 2 sectors of a municipality (data collected May–June 2020 and November–December 2020, respectively). COVID-19 restrictions in Sweden were similar throughout the data collection period, with organizations requesting workers to work from home, as per governmental recommendations. We invited all employees of these 3 organizations to participate in a questionnaire, and those responding were asked about their interest to participate in technical measurements of physical behaviors. In the present study, we only included office workers with permanent full-time contracts, who worked in day shifts and who reported to be hybrid workers. Workers in the industrial company (*N* = 101) worked in administration, sales, engineering, and information technology support. Workers in the municipality (*N* = 64) worked with tasks related to administration in sections responsible for, e.g. buildings, infrastructure, recreational areas, sport facilities, and cultural activities. All participants signed an informed consent and data collection was performed according to the Declaration of Helsinki. The Swedish Ethical Review Authority (2019-06220) approved the study.

### Descriptive characteristics

The organizations provided the researchers with personal data (gender and age) and contact information for all workers, who then received an e-mail with a personal link to a web-based questionnaire. If the questionnaire was not completed, the worker received up to 3 weekly e-mail reminders. In the questionnaire, we asked the workers about their opportunities to work remotely (“How much do you use the opportunity to work remotely during the ongoing pandemic?”). The answer was reported on a scale with 3 answer categories: *Yes, to a high or somewhat high extent*; *Yes, to some extent* and *No, not at all*. Workers replying “*No, not at all*,” were excluded from further analysis. Thus, workers who could work ‘remotely’ to some or a high extent were classified as hybrid workers. We also asked about smoking habits (smoker or non-smoker), self-reported physical activity during leisure (*physically inactive, some light physical activity, regular physical activity,* or *regular training for competitive sports*) (Grimby et al. 2015) and the number of children in the household (0–12, ≥13 years old). General health was reported using a single question from SF36 (Sullivan et al. 1995) and classified as *excellent/very good, good*, or *fair/bad*. Psychosocial factors at work (quantitative demands, influence at work, quality of leadership) were assessed using the short version of the third Copenhagen Psychosocial Questionnaire (Burr et al. 2019). A member of the research team measured the participating workers height and weight, which were then used to calculate the body mass index in kg/m^2^.

### Assessments of physical behaviors

We measured physical behaviors for 7 consecutive days around the clock, using a thigh-worn accelerometer (Axivity AX 3, Axivity Ltd., Newcastle, UK). The accelerometer was attached at the mid-front of the right thigh by a member of the research team using double-sided adhesive tape (3M, Hair-Set, Saint Paul, Minnesota, USA) and Fixomull (Fixomull BSN medical GmbH, Hamburg, Germany). During the 7 days of measurement, participants reported what time they woke up and went to bed in a diary; thus, time in bed (TiB) and time awake were identified from the diaries. For each workday, participants reported if work was performed predominantly, defined as at least 50% of the working day, at home (categorized as WFH) or in the office (categorized as WAO). The time awake was partitioned in sitting and non-sitting behaviors, including discriminating between sitting uninterruptedly for different bout durations (see below).

### Preparation of the dataset and data processing

We processed accelerometer data with the Acti4 algorithm, using a custom-made MATLAB software that can identify different types of physical behavior with good validity (Stemland et al. 2015). We excluded the first and last day of data collection for all workers to ensure that only full days with 24 h of data were included in the analysis. For each day and person, we obtained a composition containing 5 parts: TiB, non-sitting time, and sitting time in uninterrupted bouts of short (0–5 min), moderate (5–30 min), and long (>30 min) duration. Sitting time included both sitting and lying behaviors which cannot be separated by Acti4 when only a thigh accelerometer is used, while non-sitting included standing, walking, stair walking, running, and cycling. We illustrated the distributions of individual behaviors during days WAO and WFH, as well as during NWD in cumulative distribution plots, and calculated the mean time spent in each behavior for all day types. Times spent in different physical behaviors during one day are inherently co-dependent, since they add up to 24 h. Thus, if time in one behavior is reduced, time in one or more other behaviors by necessity increases. This leads to data having particular statistical properties that cannot be handled using standard analysis methods (Dumuid et al. 2020; Gupta et al. 2020). In order to properly process and analyze such “compositional” data, a specific set of techniques, Compositional Data Analysis (CoDA), has been developed. In CoDA, the compositional parts are transformed to a set of orthogonal (i.e. statistically independent) log-transformed ratios, which can be analyzed using standard statistics (Dumuid et al. 2020; Gupta et al. 2020). Thus, for each worker and day type, we transformed the composition into an orthogonal set of 4 coordinates as follows:

ILR_1_ represents the ratio of TiB to time awake. IRL_2_ represents time spent non-sitting relative to sitting time during waking hours. IRL_3_ represents the time spent in short bouts of sitting (<5 min) relative to moderate and long bouts of sitting (5–30 min and >30 min), and finally ILR_4_ represents the time spent in moderate bouts of sitting (5–30 min) relative to long bouts of sitting (>30 min). These 4 coordinates together describe the composition of behaviors during a 24-h day in a way that reflects and details the aims of the study, i.e. to understand the extent to which TiB (relative to time awake), time in non-sitting (relative to sitting), and time in sitting bouts of different durations (relative to time in bouts of other durations) differ depending on day type and gender.

### Statistical analysis

We used multilevel linear mixed models to account for the nested structure of the data by using the 3 different organizations and the workers as random effects. We ran 4 multilevel models to determine the unadjusted association between each of the 4 ILRs and day type (WAO and WFH against NWD as the reference) as a fixed effect. Gender (both the main effect and the interaction with day type) was also considered as a fixed effect and age was included as covariate. Worker and intercept were included as random effects. The interaction between gender and day type was not included in the final model if it was not significant. We also made post-hoc pairwise comparisons between day types using the Least Significant Difference test. The analyses (crude and adjusted) were conducted on workers with data for at least one day type, and missing data were assumed missing at random (MAR). Thus, we accommodated the mixed nature of the sample, in containing both workers with repeated measurements from WAO as well as WFH days, and workers who had only data from one of these day types. All but 7 workers had data from NWDs. The significance level was set at 0.05. All analyses were performed using the Statistical Package for the Social Sciences (SPSS, v 27.0, IBM, Armonk, NY, USA).

## Results

Among the 4,076 workers employed at the 3 selected organizations, 1,495 completed the questionnaire. All employees responding to the questionnaire and indicating their interest in participating in the physical behavior measurements were invited to take part (*n* = 210). Of these, we excluded 24 workers who reported to not have the possibility to work from home. In addition, we excluded the first and the last day of measurement for participating workers, as well as days with incomplete data (i.e. less than 24 h). The final study sample comprised 165 full-time hybrid office workers with, in total, 938 complete measurement days (311 WAO, 298 WFH, and 329 NWD). Thus, participants worked from home during 49% of the measured working days. Their background characteristics, stratified by gender are shown in Table 1. More than half of the population were women (*N* = 98, 59.4%), had children up to 12 years old living at home (*N* = 85, 51.5%), and reported doing regular physical activity or training for competitive sports (*N* = 114, 69.1%).

**Table 1.** Background characteristics for the hybrid office workers, stratified by gender.

	Study population (*N* = 165)		Men (*N* = 67)		Women (*N* = 98)	
	*N* (%)	M (SD)	*N* (%)	M (SD)	*N* (%)	M (SD)
Age		42.5 (10.7)		42.9 (10.5)		42.2 (10.8)
Body mass index (kg/m^2^)		25.4 (4.1)		26.3 (3.7)		24.8 (4.3)
Smoke habits (missing *n* = 1)						
Smoker	1 (0.6)		0 (0.0)		1 (1.0)	
Non-smoker	163 (98.8)		66 (98.5)		97 (99.0)	
Self-reported physical activity						
Physically inactive	9 (5.5)		4 (6.0)		5 (5.1)	
Some light physical activity	42 (25.5)		11 (16.4)		31 (31.6)	
Regular physical activity	94 (57.0)		44 (65.7)		50 (51.0)	
Regular training for competitive sports	20 (12.1)		8 (11.9)		12 (12.2)	
General Health (missing *n* = 3)						
Excellent or very good	67 (41.2)		30 (45.5)		37 (38.6)	
Good	73 (45.1)		27 (40.9)		46 (47.9)	
Fair or Bad	22 (13.5)		9 (13.6)		13 (13.6)	
Quantitative demands (scale 0–100)		51.8 (20.8)		51.9 (22.5)		51.7 (19.7)
Influence at work (scale 0–100)		53.9 (14.3)		55.0 (17.3)		53.1 (11.8)
Quality of leadership (scale 0–100)		63.6 (21.9)		63.7 (18.6)		63.5 (24.0)
Workers with children in the household						
Children from 0–12 years	85 (51.5)		35 (52.2)		50 (51.0)	
Children from ≥ 13 years	57 (34.5)		27 (40.3)		30 (30.6)	
No children	23 (14.0)		5 (7.5)		18 (18.4)	
Number of workers; number of measured days and M (SD) of days per worker for the different day types						
Work at the office	124; 311	2.5 (1.1)	52; 141	2.7 (1.2)	71; 170	2.4 (1.1)
Work from home	115; 298	2.6 (1.1)	44; 111	2.5 (1.3)	72; 187	2.6 (1.1)
Non-workdays	156; 329	2.1 (0.6)	64; 127	2.0 (0.6)	96; 202	2.1 (0.7)

*N*, number of workers; M, mean; SD, standard deviation.

### Time spent sitting, non-sitting and in bed

**Fig. 1.**
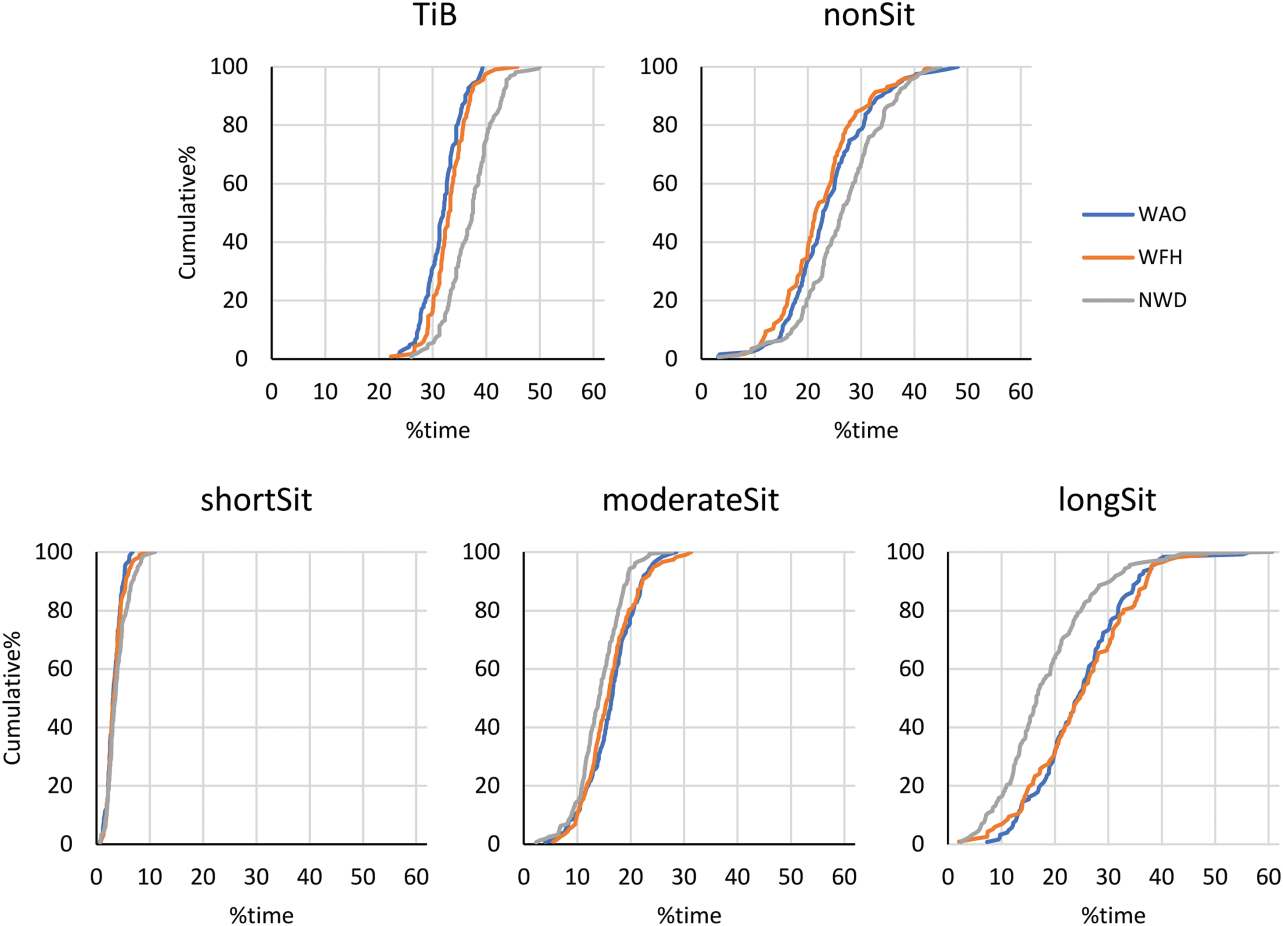
Cumulative distribution plots for the study population of time in bed (TiB), non-sitting behaviors (nonSit) and sitting time partitioned into short bouts (shortSit), moderate bouts (moderateSit) and long bouts (longSit). The x-axes show the percentage of time spent in the behavior, and the y-axes show cumulative percentages of the population. In all diagrams, data are shown for days working at the office (WAO—blue lines), days working from home (WFH—orange lines) and nonworking days (NWD—gray lines).

Cumulative distribution plots of the behaviors for each day type (Fig. 1) illustrate the dispersion among participants. We found considerable differences between participants, especially in the time spent in long bouts of sitting, which ranged from about 5% to about 60% time, and in non-sitting behaviors, where the range was 5% to 45% time. Notably, these differences between participants are, to some extent, inflated by day-to-day variability within participants. We did not specifically assess within-participant variability because data were limited at the level of individual days: only little more than 2 measurement days were, on average, available for each participant and day type (Table 1).

**Fig. 2.**
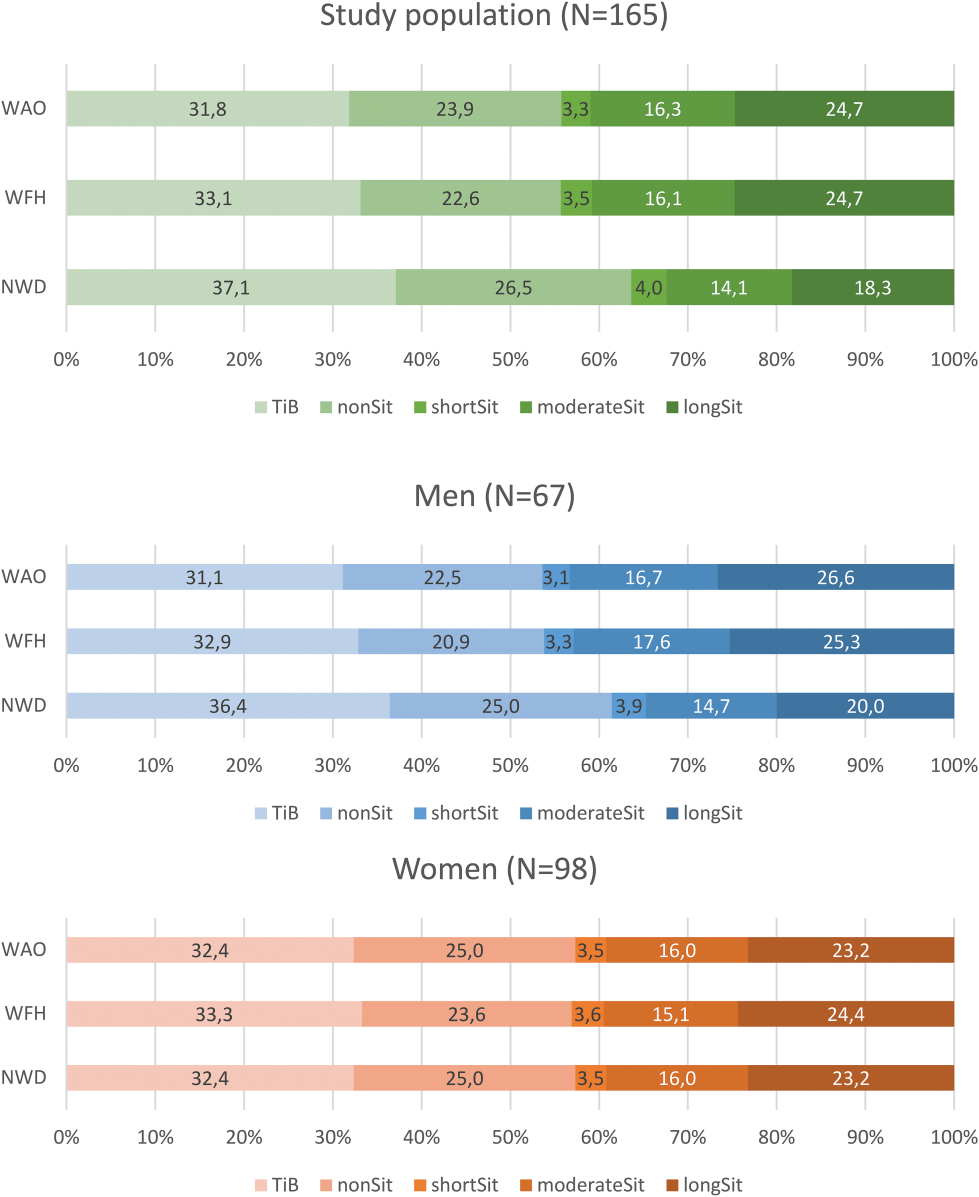
Average relative time use (percentages) in different behaviors for different day types, for the total population and the population stratified by gender.

During workdays (both WAO and WFH) workers spent, on average, ~44% (630 min) of their total 24 h sitting (short, moderate, and long bouts combined). During NWD, ~36% of the time (520 min) was spent sitting (Table 2; Fig. 2).

**Table 2.** Mean (SD) of the 5 physical behaviors, in minutes, and ILR_1-4_ according to CoDA, during days WAO, WFH, and NWD.

	Study population			Men			Women		
	WAO (*N* = 123)	WFH (*N* = 116)	NWD (*N* = 158)	WAO (*N* = 53)	WFH (*N* = 45)	NWD (*N* = 63)	WAO (*N* = 70)	WFH (*N* = 71)	NWD (*N* = 95)
TiBa	458.3 (50.6)	477.1 (50.1)	534.4 (65.8)	448.1 (50.4)	473.2 (48.5)	524.3 (76.2)	466.0 (49.6)	479.5 (51.3)	541.1 (57.2)
nonSit	344.3 (113.0)	325.1 (110.2)	381.9 (113.8)	324.2 (113.1)	301.6 (107.1)	360.4 (132.6)	359.6 (111.2)	340 (110.3)	396.2 (97.5)
shortSit^b^	47.4 (18.8)	50.6 (22.3)	57.0 (28.5)	44.1 (18.7)	48.1 (20.5)	55.6 (31.4)	50.0 (18.5)	52.2 (23.4)	57.9 (26.6)
moderateSit^c^	234.6 (69.5)	231.4 (72.1)	203.4 (60.9)	240.2 (67.9)	253.2 (76.3)	212.2 (71.4)	230.4 (70.9)	217.6 (66.3)	197.5 (52.5)
longSit^d^	355.4 (126.3)	355.8 (138.6)	263.3 (131.7)	383.5 (136.4)	363.9 (140.3)	287.5 (159.3)	334.0 (114.4)	350.7 (138.2)	247.2 (107.7)
ILR_1_	0.83 (0.20)	0.88 (0.18)	1.03 (0.24)	0.83 (0.25)	0.87 (0.19)	1.03 (0.31)	0.84 (0.17)	0.88 (0.18)	1.03 (0.18)
ILR_2_	0.68 (0.40)	0.62 (0.39)	0.88 (0.38)	0.60 (0.40)	0.54 (0.37)	0.8 (0.45)	0.74 (0.40)	0.68 (0.40)	0.93 (0.33)
ILR_3_	−1.5 (0.42)	−1.43 (0.45)	−1.17 (0.54)	−1.62 (0.44)	−1.53 (0.43)	−1.26 (0.62)	−1.41 (0.38)	−1.37 (0.45)	−1.11 (0.47)
ILR_4_	−0.28 (0.41)	−0.27 (0.50)	−0.13 (0.53)	−0.33 (0.43)	−0.23 (0.48)	−0.15 (0.63)	−0.25 (0.40)	−0.29 (0.51)	−0.11 (0.45)

Data are presented both for the total population and stratified by gender.

aTiB, time in bed; ^b^shortSit, sitting without interruption for <5 min, ^c^moderateSit, sitting without interruption for 5-30 min, ^d^longSit, sitting without interruption for >30 min. N, number of days. ILR_1_: TiB/time awake, ILR_2_: nonSit/Sit, ILR_3_: shortSit/moderateSit & longSit, ILR_4_: moderateSit/longSit.

On average, workers spent ~32% (458 min) of their total 24 h in TiB on WAO, 33% (477 min) on days WFH and 37% (534 min) during NWD. In general, men and women spent similar amounts of TiB during workdays, while men spent more time sitting and women consequently more time non-sitting, regardless of WAO or WFH (Table 2). During NWD, men spent more TiB and less time sitting in total, while the amount of time spent non-sitting was similar between men and women (Table 2).

### Effects of day type on physical behaviors

We found no significant interactions of gender with day type for any of the compositional behavior ratios (ILR_1_-ILR_4_). Analyses therefore proceeded without interaction term. After adjusting for age and using NWD as a reference, days WAO and WFH were both significantly associated with less TiB relative to awake time (ILR_1_), less non-sitting relative to sitting time (ILR_2_), less time in short sitting bouts relative to moderate and long sitting bouts (ILR_3_), and less time in moderate relative to long sitting bouts (ILR_4_) (Table 3). In other words, workdays (both WAO and WFH) were associated with less TiB and more time in sitting than NWD, and this time was to a larger extent spent in moderate and long bouts of sitting (Fig. 2).

**Table 3.** Effects of day type and gender on relative time-use in physical behaviors, after adjustment for age (*n* =165).

	TiB/ time awake (ILR_1_)			nonSit/ Sit (ILR_2_)			shortSit/ moderateSit & longSit (ILR_3_)			moderateSit/ longSit (ILR_4_)		
	B	95% CI	*P*	B	95% CI	*P*	B	95% CI	*P*	B	95% CI	*P*
**Day type**												
WAO	**−0.19** ^**a**^	**−0.23; −0.16**	**<0.01**	**−0.19** ^**a**^	**−0.26; −0.11**	**<0.01**	**−0.32**	**−0.40; 0.23**	**<0.01**	**−0.15**	**−0.24; −0.05**	**<0.01**
WFH	**−0.16**	**−0.23; −0.12**	**<0.01**	**−0.28**	**−0.35; −0.21**	**<0.01**	**−0.28**	**−0.37; 0.19**	**<0.01**	**−0.16**	**−0.27; −0.05**	**<0.01**
NWD	Ref			Ref			Ref			Ref		
**Gender**												
Women	0.01	**−**0.04; 0.07	0.62	**0.15**	**0.05; 0.25**	**<0.01**	**0.18**	**0.06; 0.30**	**<0.01**	0.03	**−**0.08; 0.14	0.59
Men	Ref			Ref			Ref			Ref		

Results from linear mixed models based on ILR.

NWD: non-working days. Significant (*P* < 0.05) effects are marked in bold.

^a^Significant difference (*P* < 0.05) in pairwise comparison of WAO and WFH. Results are adjusted for age.

The post-hoc pairwise comparisons of workdays in the adjusted models showed that, compared to days WAO, days WFH were associated with longer TiB relative to awake time (ILR_1_*P* = 0.03) and less time non-sitting relative to sitting (ILR_2_*P* = 0.02). Thus, workers stayed longer in bed and spent a higher proportion of their awake time in sedentary behavior on days WFH than when WAO. However, we did not find significant differences between WAO and WFH in ratios of time spent in short, moderate, and long bouts of sitting (ILR_3_*P* = 0.34; ILR_4_*P* = 0.78). Further, we found that women compared to men spent a higher proportion of time non-sitting relative to sitting (ILR_2_), and a higher proportion of their sitting time in short relative to moderate and long sitting bouts (ILR_3_), independent of the day type. The unadjusted models showed the same effects as the adjusted models and are therefore not shown.

## Discussion

In this study, we aimed to document and compare total sitting and the temporal distribution of sitting among hybrid office workers, using a compositional (CoDA) approach. We also investigated whether the time use patterns differed between men and women. To our knowledge, this is the first study using objective measurements to assess temporal patterns of sitting for days WAO and WFH during the pandemic.

During workdays, participants spent most of their waking hours sitting, which was distributed towards proportionally more time in longer bouts relative to shorter bouts. We found that workers spent ~44% (i.e. 10.6 h) of a workday sitting, regardless of WAO or WFH (Fig. 2). Studies conducted before the COVID-19 pandemic using thigh-worn accelerometers have reported that office workers spent about 40% of their workday sitting (Smith et al. 2015; Brakenridge et al. 2016; Healy et al. 2016), which is similar to our population. The proportion of time spent sitting for NWD (36.4%, Fig. 2) was in line or lower compared with pre-pandemic studies (Smith et al. 2015; Toledo et al. 2019; Wahlström et al. 2019), which reported workers to sit between 37% and 44% of the time.

Pairwise comparisons showed that participants were sitting more relative to non-sitting during WFH days compared to WAO days. The differences were significant, but small. Larger differences between WFH and WAO have been found in studies based on self-reported sedentary behavior (Blom et al. 2021; Koohsari et al. 2021; Stockwell et al. 2021). It is not clear whether the differences between WFH and WAO are due to different characteristics of the work environment when WFH or WAO, or due to other conditions following the pandemic restrictions. Our results disagree with those in a small Brazilian study (Brusaca et al. 2021) comparing physical behaviors in office workers before and during COVID-19. This study showed that workers spent slightly more time sitting before the pandemic, when they performed their work at the office and very rarely at home. A Swedish study by our research group (Hallman et al. 2021) on the other hand, found no differences in sitting between days WFH and days WAO during the COVID-19 pandemic. More post-pandemic research is needed on factors determining the extent of sitting when working at different locations.

We found no differences between days WFH and WAO regarding time spent in sitting bouts (short relative to moderate and long bouts, and moderate relative to long bouts). This contrasts to the pre-pandemic study (Widar et al. 2021), reporting that workers more often switched between sitting and standing when WFH than when WAO. It is likely that WFH prior to the pandemic to a greater extent than during the pandemic would have been confined to work tasks particularly suited for homework, as opposed to the pandemic conditions, where restrictions implied a more restricted autonomy in selecting where to work, and hence which tasks to perform at home. As for the total extent of sitting, more research is needed on determinants of the sitting pattern when working at different locations.

Days WFH were associated with 19 minutes more TiB than days WAO, and this difference was significant (Table 3). This result agrees with previous studies performed in Sweden and Brazil during the COVID-19 pandemic, indicating that workers spent longer TiB on days WFH than on days WAO (Brusaca et al. 2021; Hallman et al. 2021). It also agrees with a recent study from Singapore (Massar et al. 2022), reporting that sleeping time shortened when participants returned to WAO or to school after the lockdown. The reason for workers spending more time in bed when WFH may be that less time is needed for commuting, and, thus, the morning routine can be more relaxed during WFH days.

We found that women spent more time non-sitting relative to sitting than men, independent of day type, and even more time in short sitting bouts relative to moderate and long bouts. Our results generally agree with the findings in a pre-COVID study by Johansson et al. (2020), where women were sitting less at work than men. Sweden is one of the most equal countries in the world, but women still take more responsibilities for family care and household work than men (Flor et al. 2022). Thus, a possible explanation for the difference between women and men may be different work tasks (Appel-Meulenbroek et al. 2022) differences in the extent of household labor (Oslawski-Lopez 2016) and that restrictions during the pandemic may have influenced the physical behavior for women and men differently (Clemes et al. 2014). These differences are even sensitive to the cultural context (Brusaca et al. 2022; Flor et al. 2022) and we recommend future studies to investigate differences in behavior between countries, which may be considerable.

Of note, we investigated the total time awake in the present study and did not distinguish between work and leisure. We merged work and leisure because days WFH may be particularly difficult to partition into periods of (paid) work and non-work. Therefore, a comprehensive 24-h analysis of physical behaviors that does not attempt to accomplish a distinction is justified.

After the COVID-19 pandemic, hybrid work models have become more prevalent in many organizations (Chafi et al. 2021; Gilson et al. 2022; Office for National Statistics 2022). Therefore, it is of great interest to understand how these new occupational conditions are practiced, whether hybrid work is associated with health issues in certain groups, and what work environmental aspects to consider when making policies for hybrid work models. Some recommendations for sitting and sleep time are available (Bull et al. 2020; Chaput et al. 2020), but they are mainly based on questionnaire data and do not distinguish between WAO and WFH. While a review (Ekelund et al. 2019) found a significantly increased risk for mortality when sitting more than 9.5 h per day, and the Canadian 24-h movement guidelines (Ross et al. 2020) recommend sitting no more than 8 h per day, there are still not sufficient evidence to allow a quantitative threshold for time spent sitting, based on directly measured data. The present guidelines mainly consider the total amount of time spent sitting or standing and are more vague when it comes to recommending how often a person should break up sitting or alternate between postures. As an example, a European guideline for standing behaviors at work states that an optimal composition of time spent sitting, standing and being active is 60%/30%/10%, and that alternations between the 3 behaviors should occur “as much as possible,” and with no period of uninterrupted standing being longer than 1 h (Peereboom et al. 2021). Although working from home only resulted in a small increase in sitting time in the present study compared to working at the office, our hybrid office workers spent a considerable proportion of time in sitting during workdays compared to non-workdays, and extensive sitting may lead to negative health consequences. Intervention studies have so far not been designed to reduce sitting time specifically for hybrid workers, but such interventions are likely of importance in the future, since the occurrence of hybrid office work has increased profoundly in industrialized countries after the pandemic.

Strengths of the present study are the relatively large study population, considering that data was collected using technical measurements, and that it included office workers in a hybrid arrangement from both private and public sectors. In Sweden, recommendations were issued by the Public Health Agency to diminish the spread of the corona virus and workers were strongly recommended to work from home. Since working from home was not strictly mandatory, we could collect data on the same individuals both when WAO and WFH. Thus, while data were collected under conditions that may not apply entirely to a working life free of COVID-19 restrictions, a major part of the office workers in our sample were likely comfortable with a hybrid model, since they practiced that to a considerable extent already prior to the pandemic. Another strength in the study design is the use of CoDA to process 24-h accelerometry data.

A limitation is that we did not have access to data on work tasks performed during WFH and WAO. We also have limited knowledge on whether the measured physical behaviors were largely controlled by the workers themselves, including whether they were encouraged by their organizations or mass media to take breaks from sitting and to be physically active during the pandemic. Even though we trust our results to be fairly representative and generalizable to post-pandemic conditions, both results and effect sizes need to be confirmed in larger study populations in a post-pandemic work environment. Notably, our study may have lacked power to detect effects of a relevant size, in particular for interactions between day type and gender, but methods for tracking down and interpreting power in analyses based on CoDA have not so far been developed. Also, the effect of gender on behaviors may have been affected by factors differing between men and women that were not assessed in the present study. Another limitation is that not all participants were measured during all 3 day types, i.e. WAO, WFH, and NWD. Thus, we did not have a complete repeated-measures design, and data did not allow an analysis of within-participant variability in behaviors for each of the 3 day types. However, we believe that participants were “MAR,” i.e. that the short period of measurement per worker, 7 days, led to some workers not having worked at the office any of the 7 days, even if they did so at regular intervals, while others did not work from home. Irrespective of this random event, we trust that all 165 workers in the population were hybrid workers, according to their questionnaire answer. However, we cannot exclude the possibility that workers working mainly from home, and thus having a larger probability of being measured during WFH days, had other tasks, and thus other physical behaviors, than workers being measured during WAO days.

## Conclusion

We found that hybrid office workers spent more time in bed and were sitting more during their time awake on days working from home compared to days working at the office, while the distribution of sitting time between short, moderate, and long uninterrupted bouts did not differ consistently between work locations. Sitting time increased considerably during working days compared to non-working days and was higher in men than in women. The results can support the development of organizational policies for hybrid work.

## Supplementary Material

wxad057_suppl_Supplementary_Figure

## Data Availability

The data underlying this article will be shared on reasonable request to the corresponding author.
