# Supplementary figures and images for: Hybrid office work in women and men: do directly measured physical behaviors differ between days working from home and days working at the office?
Source: Ann Work Expo Health. 2023 Oct 3;67(9):1043–55. doi: 10.1093/annweh/wxad057 (PMC10683849; doi:10.1093/annweh/wxad057)

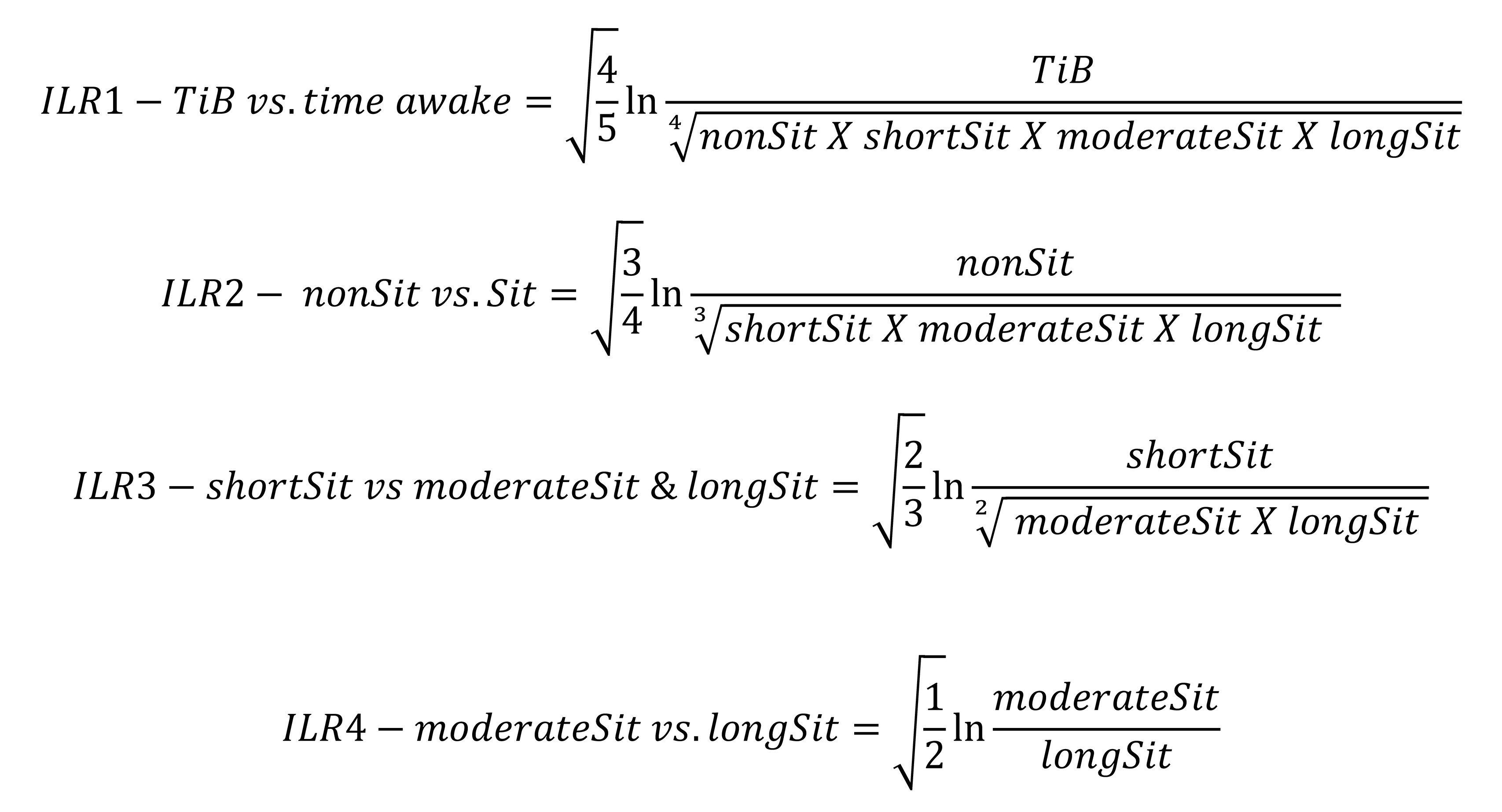

Supplement: wxad057_suppl_Supplementary_Figure [file wxad057_suppl_supplementary_figure.jpeg]
